# Supplementary material for: The association between depression and addictive social media use during the COVID-19 pandemic: The mediating role of sense of control
Source: PLoS One. 2023 Sep 8;18(9):e0291034. doi: 10.1371/journal.pone.0291034 (PMC10490948; doi:10.1371/journal.pone.0291034)
Supplement: Attachment — Submitted filename: Response to Reviewers.docx [file pone.0291034.s002.docx]

**Response to comments following review.**

**Editorial comments.**

1. The paper has been revised according to the journal’s style guidelines **–** headings, file naming etc.
2. A deidentified dataset is now made available as a supplementary file.
3. The fact that written informed consent was obtained in clarified in the methods section.
4. A title page is included in the main document.

**Reviewer 1 comments.**

1. We have modified the title as follows: we trust this change will find your positive approval. The association between depression and addictive social media use during the COVID-19 pandemic: The mediating role of sense of control.
2. The abstract has been expanded by adding the following details as you requested: setting, design, subjects, sampling technique, and statistical results.
3. Introduction:

- the Intro has been edited as far as possible with the view to shorten it. Paragraphs have also been abbreviated to ensure none are longer than 5 sentences at the most. To truncate this any further would result in a substantial loss of essential information.
- In sum, the Introduction is laid out as follows: the mental health consequences of COVID are presented, including specifically the rise in addictive SMU; addictive SMU is then presented (description of features, associated mental health consequences, and with specific reference to COVID); sense of control is introduced and associated literature reviewed with the specific intention to introduce the proposition for its likely mediational properties; and finally, a theoretical framework. We regard this format as logical and the best approach in constructing the argument for the research question and its hypotheses.
- Any use of the ‘we’ pronoun has been removed.

4. Method:

- The following edits and/or additions have been made in concurrence with your requests: method section now commences with description of the study design, sampling technique is included, description of the targeted classes from students were drawn.
- The inherent limitation of selective reporting and social desirability when using self-report measures is acknowledged and its potential consequences discussed, at length, in the limitations section. This is not uncommon; indeed, it is the norm in psychological research. However, participants were provided with information in the participant info form, among others, of anonymity of data, protection of data, and who would have access to the data they reported. These measures were designed to assure participants and ensure accurate reporting of data, as far as this is possible within the confines of a cross-sectional study.
- The 2-item scale has been extensively used in psychological research, including in relation to COVID research. It has been shown to be a valid measure of the construct. These studies are referenced in substantiation of its use.

5. Results: The gender distribution of the sample mirrors the gender distribution of the sampling population and is therefore a valid representation of the population. This is not the result of selective sampling. Moreover, gender has been included as covariate in the mediation model to account for any potential variation, if any.
6. Discussion: This section has been edited and modified substantially with the view to truncate it as well as construct a thorough interrogation of the results. The only consideration here is that there are very few previous studies – barring one – that serve as a neat point of comparison for our results (thus enabling comparative discussion, of similarities and contradictions etc). In fact, the study’s research question – confirmation or disconfirm of the hypothesized mediation model is discussed in relation to preceding literature and the I-PACE model, which informed the research question. We believe this to be a prudent course of action in constructing the discussion.
7. Conclusion: paragraph written.
8. Implications: paragraph written.

**Reviewer 2 comments:**

1. Discussion: This section has been edited and modified substantially with the view to truncate it as well as construct a thorough interrogation of the results. The only consideration here is that there are very few previous studies – barring one – that serve as a neat point of comparison for our results (thus enabling comparative discussion, of similarities and contradictions etc). In fact, the study’s research question – confirmation or disconfirm of the hypothesized mediation model is discussed in relation to preceding literature and the I-PACE model, which informed the research question. We believe this to be a prudent course of action in constructing the discussion.
2. Conclusion: paragraph written.
