# Supplementary material for: The association between depression and addictive social media use during the COVID-19 pandemic: The mediating role of sense of control
Source: PLoS One. 2023 Sep 8;18(9):e0291034. doi: 10.1371/journal.pone.0291034 (PMC10490948; doi:10.1371/journal.pone.0291034)
Supplement: Attachment — Submitted filename: Response to reviewer comments.docx [file pone.0291034.s003.docx]

Response to reviewer comments

Reviewer 1

Comment: How the researchers conducted snowball sampling?

Response: A sentence has been added to clarify how this was achieved on pages 8-9.

Comment: Heading "materials and methods" should be replaced with materials and method.

Response: Done.

Comment: In discussion : replace "the potential mediational role of variation in sense of control" with the potential mediating role of sense of control
Response: Done.

Comment: Implications are still very weak- how this study will add to the clinicians.

Response: This has been substantially expanded across pages 18-19.

Reviewer 3.

Comment: In the part of Abstract, A combination of convenience and snowball sampling were used. However, in the part of Procedure and participants, his study employed a convenience sampling approach to collect data from college aged
participants.
Response: the text in the Method section has been amended to include this omission.

Comment: The first sentence of the discussion should be followed by the second paragraph of the discussion.
Response: Done.

Comment: There are no discussion on the relationship between depressive symptoms and SMU in the third paragraph of the discussion.

Response: Paragraph 2 on page 14 has been expanded substantially to expand on the discussion related to the association of these variables.

Comment: Why Table 1 and Table 2 were listed stratified by country, but mediation analyses were not stratified by country

Response: Country-specific descriptive data is provided for descriptive purposes only. Mediation analyses were conducted separately as the UAE sample would be too small on which to run a sufficiently powered analysis. Thus, a mediation analysis on the total sample was deemed to be the most prudent approach, while the descriptive data relating to each country may be of interest to some readers.
